# Enhancing Tissue Integration and Reducing Inflammation in Silicone and Human Acellular Dermal Matrix Implants via Vacuum Plasma Treatment

**DOI:** 10.3390/ijms26125854

**Published:** 2025-06-18

**Authors:** Kyung Bae Chung, Young In Lee, Jihee Kim, Ngoc Ha Nguyen, Yoo Jin Kim, Inhee Jung, Jeonghoon Lee, Hyun-Jeong Jeon, Youbong Lim, Sung Jun Lee, Ju Hee Lee

**Affiliations:** 1 Department of Dermatology, Cutaneous Biology Research Institute, Yonsei University College of Medicine, Seoul 03722, Republic of Korea; chungkyungbae@yuhs.ac (K.B.C.); ylee1124@yuhs.ac (Y.I.L.); mygirljihee@yuhs.ac (J.K.); nguyenngocha7996@gmail.com (N.H.N.); 2 Scar Laser and Plastic Surgery Center, Yonsei Cancer Hospital, Yonsei University College of Medicine, Seoul 03722, Republic of Korea; 3 Department of Dermatology, Yongin Severance Hospital, Yonsei University College of Medicine, Yongin-si 16995, Republic of Korea; 4 Department of Dermatology, University of Medicine and Pharmacy, Ho Chi Minh City 17000, Vietnam; 5 Global Medical Research Center, Seoul 137-865, Republic of Korea; kyj@gmrc.co.kr (Y.J.K.); ihjung@gmrc.co.kr (I.J.); 6 Plasmapp Co., Ltd., Seongnam-si 13494, Republic of Korea; hjjeon@plasmapp.com (J.L.); jhlee@plasmapp.com (H.-J.J.); yblim@plasmapp.com (Y.L.); 7 Liting Plastic Surgery, Seoul 06035, Republic of Korea; justdisj@naver.com

**Keywords:** plasma, biocompatibility, silicone, tissue integration, inflammation, wound healing, reconstructive surgery

## Abstract

Plasma, an ionized gas composed of charged particles, has shown therapeutic potential in enhancing biological processes such as wound healing and tissue integration. Implants, such as silicone and human acellular dermal matrix (hADM), are commonly used in reconstructive surgery, but improving their biocompatibility and integration remains a challenge. This study investigated the effects of vacuum plasma treatment on silicone and hADM implants using an in vivo rat model. Plasma-treated and untreated implants were inserted subcutaneously, and tissue samples were collected at 1, 4, and 8 weeks post-implantation. Histological and immunohistochemical analyses were performed to assess inflammation, cellular infiltration, collagen formation (neocollagenesis), and angiogenesis. Results showed that plasma-treated silicone and hADM implants had significantly reduced capsule thickness at weeks 4 and 8 compared to untreated controls, indicating a lower chronic inflammatory response. Plasma treatment also promoted greater fibroblast infiltration and enhanced neocollagenesis within the hADM implants. Furthermore, immunohistochemical staining revealed a notable increase in blood vessel formation around and within the plasma-treated hADM implants, suggesting improved vascularization. In conclusion, vacuum plasma treatment enhances the biocompatibility and tissue integration of implants by reducing inflammation and promoting cellular and vascular responses, offering promising potential for improving outcomes in reconstructive surgery.

## 1. Introduction

Plasma, an ionized gas composed of electrically charged particles, has emerged as a therapeutic tool across various medical fields [1,2]. Studies have demonstrated plasma’s beneficial effects on biological processes such as wound healing, angiogenesis, hair growth, and stem cell activation, suggesting its positive impact on skin health [3,4,5,6,7,8]. Additionally, plasma has been shown to improve the performance of medical implants, including dental and bone materials, by enhancing cell adhesion and providing antimicrobial properties [9,10,11]. Human acellular dermal matrix (hADM) and silicone implants are commonly used in reconstructive and cosmetic surgeries. Silicone implants are widely used in breast augmentation and reconstruction surgeries to restore volume. In contrast, hADM is a biological collagen matrix devoid of antigenic properties, designed to preserve structural integrity, promote healing, and stimulate the body’s tissue growth due to its excellent biocompatibility [12]. Since hADM lacks antigenic properties, it evokes minimal tissue reactions, thus demonstrating exceptional biocompatibility without triggering immune rejection [13]. Silicone implants and hADM can be used together to reduce capsular contracture, a common complication associated with silicone implants [14,15]. Furthermore, hADM complements silicone implants by providing structural support, reducing complications, and enhancing the body’s response to foreign materials during reconstructive and cosmetic procedures. Recent studies have shown that the combination of plasma treatment and hADM, specifically through the application of vacuum or atmospheric pressure plasma on hADM, leads to improved biocompatibility and biointegration [16]. It has also been suggested that hADM, frequently used in reconstructive surgeries, can be further optimized by plasma treatment to enhance its properties [16]. In this study, we investigate the effects of vacuum plasma treatment on both silicone implants and hADMs with a focus on biocompatibility and biointegration, using an in vivo model. To assess how plasma-treated hADMs contribute to improved biocompatibility, we evaluated cellular changes and integration at the tissue level.

## 2. Results

### 2.1. Plasma Does Not Alter the Acute Immune Response Post-Implantation

**Figure 1:**
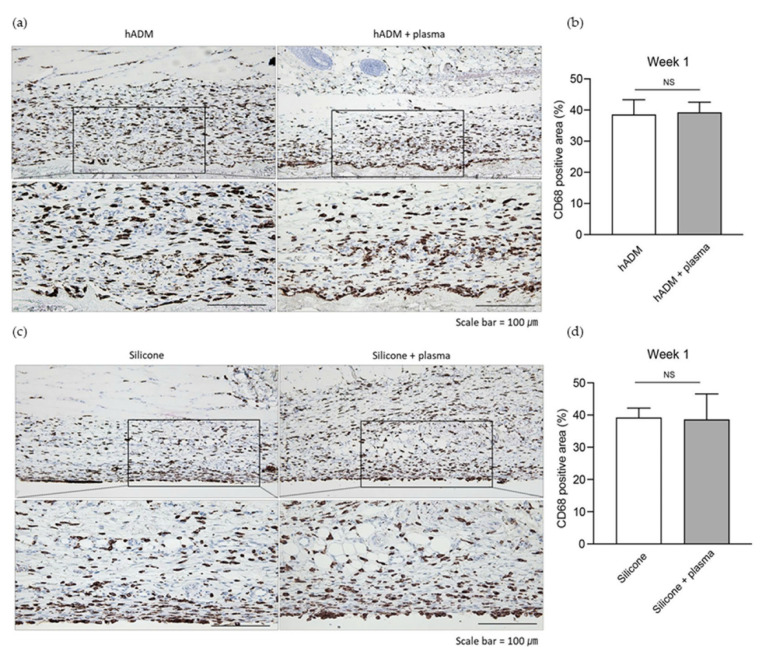
Evaluation of acute inflammatory cell infiltration following hADM implantation. (**a**) Immunohistochemical analysis of CD68-positive cells to assess acute inflammation in hADM-implanted skin. Scale bars, 100 μm. (**b**) Quantification of the CD68-positive area as a percentage within a defined region of the hADM implant. (**c**) Immunohistochemical analysis of CD68-positive cells in silicone-implanted skin. Scale bars, 100 μm. (**d**) Quantification of the CD68-positive area as a percentage within a defined region of the silicone implant. Data are presented as means ± standard deviation (paired *t*-test; NS, not significant).

To assess the early host response to the implanted materials, we evaluated acute inflammation one week after implantation. Macrophage infiltration is a key component of the initial immune response to foreign bodies and, thus, serves as an indicator of acute inflammation following biomaterial implantation [17,18]. One week after the implantation of hADM and silicone into dorsal rat skin via a 10 mm incision, acute inflammation was evaluated using CD68 staining, which labels macrophages [18]. Histological analysis revealed no significant differences in the inflammatory response between plasma-treated and untreated hADM implants, with CD68-positive areas remaining comparable (38.5 ± 4.8 vs. 39.2 ± 3.3%) (Figure 1a,b). Similarly, no significant differences in inflammation were observed between plasma-treated and untreated silicone implants (39.2 ± 3.0 vs. 38.6 ± 8.0%) (Figure 1c,d).

### 2.2. Capsule Thickness Around Implanted Silicone and hADM

**Figure 2:**
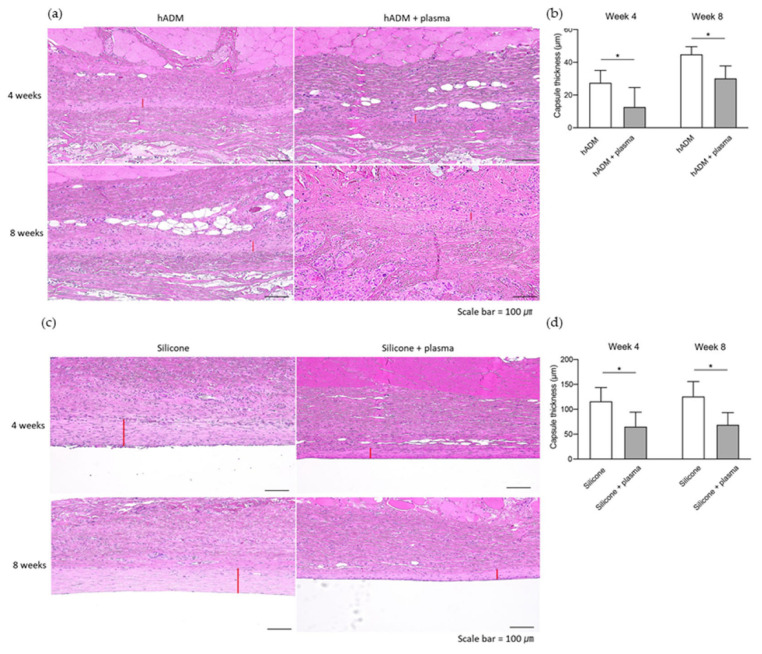
Measurement of capsule thickness surrounding implanted hADM and silicone. (**a**) Histological examination showing capsule formation around the hADM implant. Scale bars, 100 μm. (**b**) Quantitative measurement of capsule thickness in tissue surrounding hADM implants. (**c**) Histological examination showing capsule formation around the silicone implant. Scale bars, 100 μm. (**d**) Quantitative measurement of capsule thickness in tissue surrounding silicone implants. The locations of the measured capsule thicknesses are indicated by red lines in histological images. Data are presented as means ± standard deviation (paired *t*-test; * *p* < 0.05).

Following the acute phase, both silicone and hADM implants were well tolerated, showing good biocompatibility without signs of inflammation or infection. To assess chronic inflammation related to the implants, capsule thickness was measured (Figure 2a,c). As anticipated, the silicone implants exhibited a thicker capsule compared to hADM implants. Plasma-treated hADM implants displayed a marked reduction in capsule thickness compared to untreated hADM implants (Figure 2b). This reduction was evident at both week 4 (27.7 ± 7.4 vs. 12.9 ± 11.7 μm) and week 8 (45.0 ± 4.5 vs. 30.3 ± 7.4 μm), indicating that plasma treatment can mitigate capsular formation. Among the silicone implants, plasma treatment also resulted in reduced capsule thickness at both week 4 (116.6 ± 27.0 vs. 65.7 ± 28.6 μm) and week 8 (126.2 ± 29.6 vs. 69.5 ± 23.8 μm) (Figure 2d).

### 2.3. Fibroblast Infiltration and Neocollagenesis in hADM

**Figure 3:**
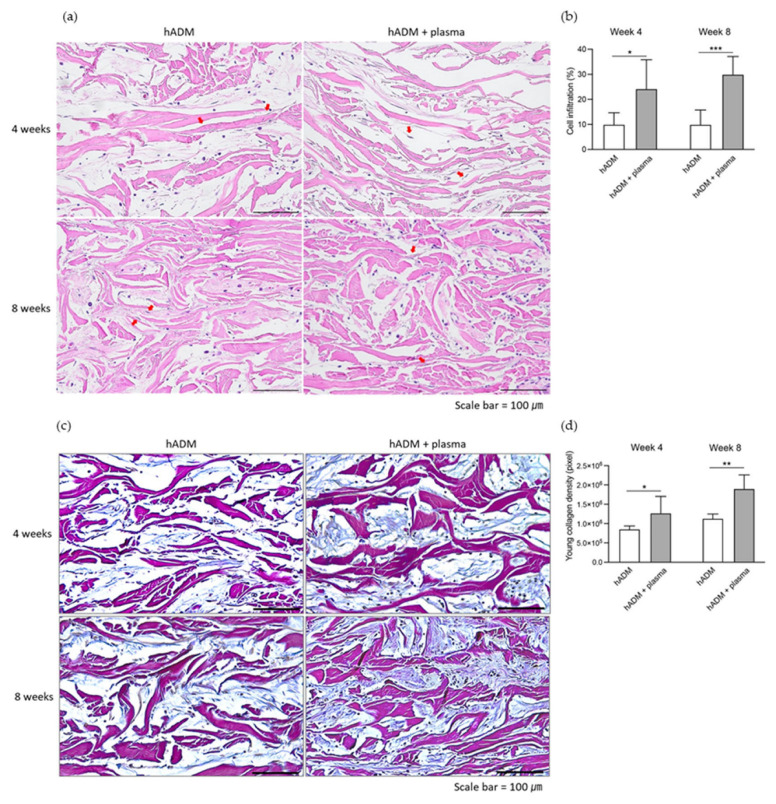
Cellular infiltration and neocollagenesis in plasma-treated hADM. (**a**) Histological images showing cellular infiltration within the hADM implant. Red arrows indicate cellular infiltration within the hADM implant. Scale bars, 100 μm. (**b**) Quantitative comparison of cellular infiltration between plasma-treated and untreated hADM implants. (**c**) Immunohistochemical staining for neocollagen using Herovici stain. Scale bars, 100 μm. (**d**) Quantification of neocollagen formation, represented by pixel count of neocollagen in the same microscopic field. Data are presented as means ± standard deviation (paired *t*-test; * *p* < 0.05, ** *p* < 0.01, *** *p* < 0.001).

Given the role of hADM in neocollagenesis and tissue repair, fibroblast infiltration and new collagen production were assessed. Cellular infiltration within hADM implants was measured to evaluate fibroblast activity. Plasma-treated hADM implants demonstrated a significant increase in cellular infiltration compared to untreated hADM implants at both week 4 (24.2 ± 11.6 vs. 10.0 ± 4.7%) and week 8 (30.0 ± 7.2 vs. 9.9 ± 5.8%) (Figure 3a,b). These infiltrating cells contributed to new collagen production. Herovici staining, used to detect newly synthesized collagen, revealed a significant increase in collagen production in plasma-treated hADM implants, with higher levels at week 4 (1.27 × 10^6^ vs. 0.86 × 10^6^ pixels) and week 8 (1.90 × 10^6^ vs. 1.13 × 10^6^ pixels) (Figure 3c,d).

### 2.4. Neovascularization Within and Around hADM

**Figure 4:**
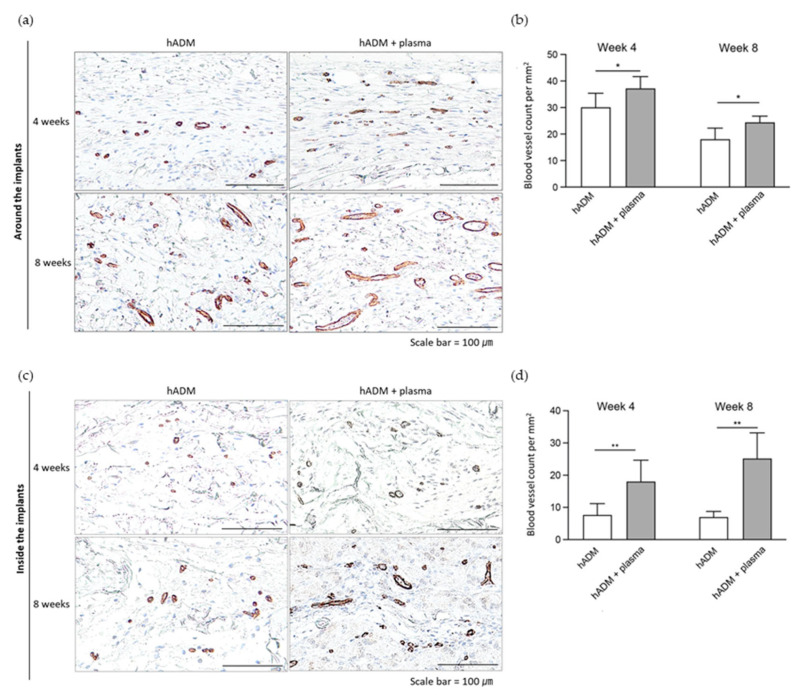
Blood vessel density surrounding and within the implants. (**a**,**c**) Immunohistochemical staining for blood vessels using CD31 around and inside the hADM implants, respectively. Scale bars, 100 μm. (**b**,**d**) Quantification of blood vessels around and inside the hADM implants, respectively. Data are presented as means ± standard deviation (paired *t*-test; * *p* < 0.05, ** *p* < 0.01).

To evaluate neovascularization within hADM implants, immunohistochemical analysis with the CD31 marker was conducted. CD31, also known as platelet endothelial cell adhesion molecule-1 (PECAM-1), is a widely recognized marker for endothelial cells and is commonly used to assess angiogenesis due to its high expression at endothelial cell junctions during new blood vessel formation [19,20]. Both the periphery and interior of the hADM implants showed an increase in endothelial cell presence, with vessel maturation and an enlargement of the lumen size by week 8 (Figure 4a,c). As shown in Figure 4b, d, the density of blood vessels was significantly higher in the plasma-treated group compared to the untreated group at week 4, with a notable difference both around the hADM (30.2 ± 5.3 vs. 37.3 ± 4.4 per mm^2^) and within the hADM (7.7 ± 3.5 vs. 18.1 ± 6.6 per mm^2^). By week 8, neovascularization in plasma-treated hADMs surpassed that in the untreated group, with higher counts both around the hADM (18.1 ± 4.1 vs. 24.5 ± 2.3 per mm^2^) and within the hADM (7.1± 1.7 vs. 25.2 ± 7.9 per mm^2^).

**Figure 5:**
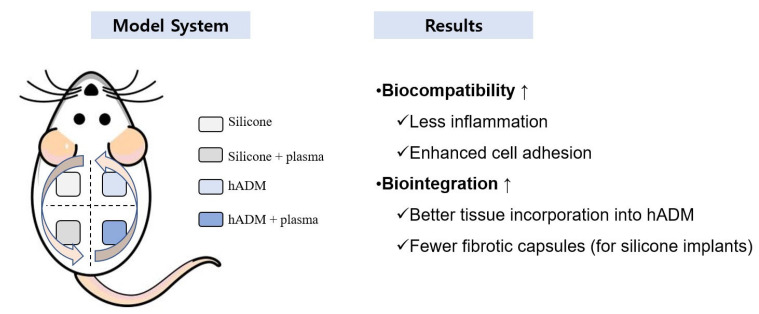
Schematic summary of the experimental model and outcomes of vacuum plasma treatment on silicone and hADM implants. The **left** side of the figure illustrates the model system, where plasma-treated and untreated silicone and hADM implants were subcutaneously implanted into four quadrants on the dorsal side of a rat. The **right** side summarizes the key findings of this study, and the symbol “↑” denotes enhanced biocompatibility and biointegration following plasma treatment.

Figure 5 summarizes both the methodology and the main outcomes of this study.

## 3. Discussion

Plasma technology has demonstrated diverse effects in the medical field, including applications in skin regeneration, wound healing, and disinfection. Beyond direct use on or within the human body, plasma can also be employed to treat the surface of medical devices and materials [8,9,10], including dental and orthopedic implants, by enhancing cell adhesion and providing antimicrobial effects. In this study, we investigated the effects of vacuum plasma treatment on silicone and hADMs. Our findings provide key insights into the potential benefits of plasma treatment for medical implants. Most notably, plasma-treated silicone and hADMs exhibited improved biocompatibility, as demonstrated by a significant reduction in capsule thickness and increased cellular infiltration in the hADMs. These results suggest that plasma treatment may enhance the integration of both materials into host tissues, reducing chronic inflammation and promoting tissue repair. Histological analysis revealed a marked reduction in capsule thickness around plasma-treated implants at 4 and 8 weeks post-implantation compared to untreated controls. This is particularly important, as thinner capsules are typically associated with a lower risk of chronic inflammatory responses and better overall biocompatibility. Plasma treatment likely improves surface properties, reducing inflammatory involvement, a finding consistent with previous studies on plasma-treated dental materials.

Additionally, plasma-treated hADMs demonstrated significantly greater fibroblast infiltration and enhanced neocollagenesis, indicating improved tissue integration and regenerative capacity [16]. Increased neovascularization was also observed, with a substantial rise in the formation of blood vessels both within and surrounding the plasma-treated hADMs. This suggests that plasma treatment may expedite the establishment of a blood supply, which is crucial for the survival and integration of implanted tissues [21]. These findings align with earlier research showing the positive impact of plasma treatment on biomaterials, enhancing cell adhesion and promotion of healing [1,9,10,11]. The reduction in capsule thickness is consistent with studies that suggest plasma-treated implants are less prone to inducing fibrotic responses, a common complication with many synthetic and biological implants [22]. Furthermore, the increased fibroblast infiltration and neocollagenesis observed in this study corroborate previous findings that highlight the role of plasma in promoting cellular activity, including proliferation and extracellular matrix production. Interestingly, the extent of neovascularization observed in this study equals or exceeds what is typically reported, suggesting that the plasma treatment parameters used here may be particularly effective in promoting angiogenesis [16,21,23]. This enhanced vascularization could have significant implications for improving the integration and function of hADMs, especially in reconstructive surgery, where early blood supply is vital for tissue survival.

The clinical implications of these findings are considerable. Plasma-treated hADMs and silicone implants could be particularly beneficial in reconstructive surgeries involving large tissue defects or areas prone to chronic inflammation [24,25,26]. The ability of plasma treatment to reduce capsule formation around implants could minimize complications such as implant rejection or the need for revision surgeries. Additionally, plasma treatment may improve outcomes in breast reconstruction surgeries, where both silicone and hADM are commonly used, by enhancing implant integration and reducing inflammatory responses. The substantial increase in neovascularization observed in plasma-treated hADMs may improve graft survival, particularly in areas with compromised blood supply. This could potentially expand the use of hADMs in more challenging surgical environments, improving outcomes in patients with complex reconstructive needs. The potential for plasma treatment to be applied to other biological and synthetic implants presents promising avenues in various medical fields, including orthopedics, dentistry, and cardiovascular surgery. In these areas, improved implant integration and long-term stability are critical for patient outcomes.

This study has several strengths, including the use of a well-controlled in vivo model and comprehensive analyses that assessed multiple indicators of biocompatibility and tissue integration. The histological and immunohistochemical methods employed provided robust data on the cellular responses to plasma-treated silicone and hADMs, deepening our understanding of the underlying mechanisms involved. However, there are some limitations to consider. The use of a rat model, though useful for initial investigations, may not fully replicate human biological environments. The relatively short study duration (up to 8 weeks) limits the ability to assess the long-term effects of plasma treatment on hADM integration. Future studies should extend the duration of experiments and explore the effects of plasma treatment in larger, more clinically relevant animal models. Additionally, the study focused on a specific plasma device and treatment protocol. Variations in plasma parameters, such as treatment duration, intensity, and gas composition, could lead to different outcomes. Further research is necessary to optimize plasma treatment protocols for different types of implants and clinical applications. Moreover, the molecular mechanisms responsible for the positive effects observed in plasma-treated hADMs remain to be identified.

Given the promising results of this study, several future research directions are recommended. First, long-term studies investigating the effects of plasma treatment on silicone and hADM integration, particularly in larger animal models that more closely mimic human physiology, are essential. Such studies could provide more definitive evidence of the clinical potential of plasma-treated hADMs. Second, exploring different plasma treatment parameters could help optimize the process for various clinical applications. For example, studies could examine how varying the duration or intensity of plasma treatment impacts tissue integration and biocompatibility, or whether different plasma gases yield distinct effects on hADMs. Third, the mechanisms underlying the enhanced neovascularization observed with plasma-treated hADMs warrant further investigation. A deeper understanding of how plasma treatment promotes angiogenesis at the molecular level could lead to more targeted therapies for improving implant integration. Lastly, clinical trials are necessary to determine the safety and efficacy of plasma-treated hADMs in human patients. These trials should assess both short-term implant integration outcomes and long-term durability and function.

In conclusion, this study provides compelling evidence that vacuum plasma treatment significantly enhances the biocompatibility and integration of silicone and human acellular dermal matrices. By reducing capsule thickness, increasing fibroblast infiltration and neocollagenesis, and promoting neovascularization, plasma treatment presents a promising approach for improving the performance of hADMs in reconstructive surgery and other medical applications. These findings contribute to the growing body of literature supporting the use of plasma technology in regenerative medicine and emphasize the need for further research to fully realize its clinical potential.

## 4. Materials and Methods

### 4.1. Device Setup and Plasma Treatment

**Figure 6:**
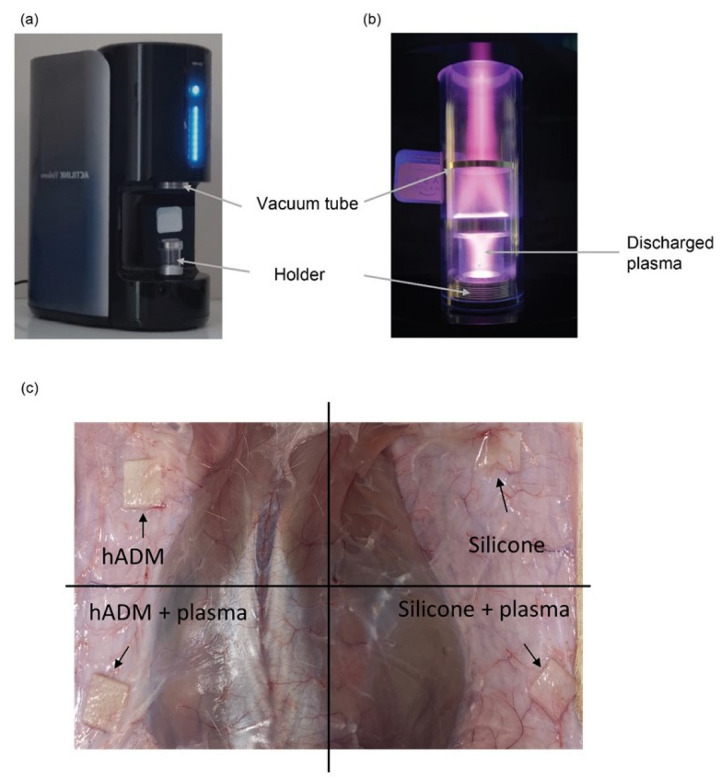
Plasma treatment and implantation setup in a rat model. (**a**) Image of the plasma activation device (ACTILINK system, Plasmapp Co., Ltd., Seoul, Republic of Korea) used to treat the surface of silicone and hADM implants. The system includes a vacuum tube and a specimen holder where the implant material is placed for treatment. (**b**) Photograph showing the device in operation. Plasma is generated under vacuum conditions and discharged within the enclosed chamber to uniformly treat the surface of the material. (**c**) Representative image of the dorsal skin of a rat showing the subcutaneous implantation of four different materials: untreated hADM (**top left**), untreated silicone (**top right**), plasma-treated hADM (**bottom left**), and plasma-treated silicone (**bottom right**). The implants were inserted into four quadrants, and the black arrows indicate the precise positions of each material under the skin.

A plasma activator (ACTILINK system, Plasmapp Co., Ltd., Seoul, Republic of Korea) equipped with a holder was used for the plasma treatment in this study (Figure 6a). A sheet-type silicone (KEOSAN, KEOSAN Trading, Seoul, Republic of Korea) or hADM (Megaderm, L&C Bio Co., Ltd., Seoul, Republic of Korea) was placed in the holder. The holder, containing the silicone or hADM, was positioned on the plasma activator and electrically connected to the ground electrode of the device. Once the process commenced, a tube was lowered from the top of the device to isolate the holder from the outside air, preventing any external air from entering. A vacuum pump then reduced the pressure inside the tube and holder to less than 10 torr. In this vacuum state, high-voltage power (a frequency of 100 kHz and a voltage of approximately 2 kV) was applied to the powered electrode placed on the top of the tube, which was about 10 cm away from the silicone or hADM. The plasma was discharged inside the tube by generating a strong electric field between the powered electrode and ground electrode. During the 30 s plasma discharge, the surface of the silicone or hADM was uniformly exposed to the plasma (Figure 6b). In this process, no additional gas is necessary for plasma discharge.

### 4.2. In Vivo Study

#### 4.2.1. Experimental Animals

Six-week-old male Sprague Dawley rats were obtained from the Orient Bio animal center (Seongnam-si, Republic of Korea). All experimental procedures were approved by the Institutional Animal Care and Use Committee of Yonsei University College of Medicine (IACUC No. 2023-0158), and the experiments were conducted in compliance with the National Institutes of Health (NIH) Guide for the Care and Use of Laboratory Animals. The rats were housed in cages under controlled conditions at 24 °C ± 0.5 °C, 55–65% humidity, and a 12 hr light/dark cycle. Food and water were provided ad libitum. Animals acclimated to the above environment for approximately one week before surgery.

#### 4.2.2. Experimental Design

A total of 18 animals were used in the experiment, and both silicones and hADM implants from four groups (control, untreated; experimental, plasma-treated) were implanted in four different areas on the back of each rat (Figure 6c). To minimize variation, the implant positions (left front, left back, right front, and right back) were rotated among 4–5 rats. Sacrifices were performed at 1, 4, and 8 weeks post-implantation, based on the analysis requirements. The silicone and hADM implants were prepared in sizes of 10 × 10 × 1 mm, with the experimental groups undergoing plasma treatment using the ACTILINK system (Figure 6b). Prior to implantation, the hADMs were hydrated in saline solution for 30 min. The rats were anesthetized with isoflurane (Hana Pharm Co., Ltd., Seoul, Republic of Korea), and the backs were shaved. After disinfecting the implant site with povidone-iodine (Firson Co., Ltd., Cheonan-si, Republic of Korea), a 10 mm incision was made, and the silicone and hADM implants were carefully placed subcutaneously using forceps. The incision was then sutured, and the site was disinfected again with povidone-iodine to minimize the risk of infection. At the end of the experiment, the rats were euthanized using CO_2_, and the silicone, hADM, and surrounding tissues were harvested for analysis.

#### 4.2.3. Tissue Section Preparation

Each rat skin tissue sample was fixed in 10% formalin (HT501128, Sigma-Aldrich, St. Louis, MO, USA) for more than 24 hr, dehydrated using a series of alcohol solutions of different concentrations (64-17-5, Merck, Burlington, MA, USA), and embedded in paraffin (1.15161, Merck). Serial sections of 5 μm thickness were prepared from the paraffin blocks. These sections were stained using hematoxylin and eosin (H&E), immunohistochemistry antibodies, and Herovici staining methods [27].

#### 4.2.4. Histological Analysis

For histological analysis, the paraffin-embedded tissues were deparaffinized and stained with hematoxylin (104302, Merck) for nuclear visualization, followed by eosin (230251, Sigma-Aldrich). The stained tissues were then dehydrated and mounted for analysis. Capsule thickness and cell infiltration were measured using the stained slides. Capsule thickness was defined as the distance (in μm) between the silicone or hADM implant and the surrounding skin tissue. Cell infiltration was quantified as the percentage of fibroblasts relative to the total cell population within the hADM.

#### 4.2.5. Immunohistochemistry

To assess the inflammatory response and angiogenesis, the expression of CD68, an inflammation marker, and CD31, an angiogenesis marker, proteins was analyzed. Deparaffinized slides underwent antigen retrieval using citrate buffer (K8005, Dako, Tokyo, Japan), followed by a 2 hr incubation in normal goat serum (ab7481, Abcam, Waltham, MA, USA). Primary antibodies Anti-CD68 (ab283654, Abcam) and Anti-CD31 (ab182981, Abcam) were applied and left to react overnight at 4 °C. The next day, a secondary antibody (anti-rabbit; k4003, Dako) was applied. After staining the nuclei with hematoxylin (SM806, Dako), the slides were mounted for analysis. The inflammatory response was quantified by measuring the total area around the implants and calculating the CD68-positive area as a percentage. Angiogenesis was assessed by counting the number of vessels per mm² in both the hADM and a region within 1 cm of the implantation site.

#### 4.2.6. Herovici Staining

For neocollagen analysis, Herovici staining was performed using the Herovici Stain Kit (Scytek, Logan, UT, USA) following the manufacturer’s instructions. After staining and dehydration, the tissue sections were mounted and photographed at 400× magnification using an optical microscope (BX43F, Olympus, Tokyo, Japan). The areas of mature collagen (purple) and neocollagen (blue) were identified and quantified from the images. Neocollagen was analyzed across the entire image using ImageJ software 1.51 (NIH, Bethesda, MD, USA), which measured the total pixel count within the same microscopic field.

### 4.3. Statistical Analysis

All data were expressed as mean ± standard deviation. Statistical analysis was conducted using SPSS software version 25.0 (IBM Corp., Armonk, NY, USA). Statistical significance was determined using Student’s *t*-test, with *p*-values less than 0.05 considered statistically significant.

## Data Availability

The data presented in this study are available on request from the corresponding author.

## Abbreviations

The following abbreviations are used in this manuscript.

hADM	Human acellular dermal matrix
CD68	Cluster of Differentiation 68 (macrophage marker)
CD31	Cluster of Differentiation 31 (endothelial cell marker)
H&E	Hematoxylin and eosin
NIH	National Institutes of Health
SPSS	Statistical Package for the Social Sciences
SME	Small and medium enterprises
KOICA	Korea International Cooperation Agency
MEF	Medical Education and Fellowship (MEF Fellowship Program)
MSS	Ministry of SMEs and Startups (Korea)
IACUC	Institutional Animal Care and Use Committee
